# Evaluation of the strategies opioid manufacturers used to recruit health professionals and encourage overprescribing: an analysis of industry documents

**DOI:** 10.1186/s12889-024-19642-z

**Published:** 2024-08-08

**Authors:** Christie Lee, Allison Tsui, Selina Xu, Dorie E. Apollonio

**Affiliations:** Department of Clinical Pharmacy, University of California, San Francisco, 530 Parnassus Ave Suite 366, Box 1390, San Francisco, CA 94143 USA

**Keywords:** Analgesics, Opioid, Inappropriate prescribing, Cancer pain

## Abstract

**Background:**

More than 263,000 individuals died due to prescription opioid misuse between 1999 and 2020. Between 2013 and 2015 alone, pharmaceutical companies spent over $39 million to market opioids to over 67,000 prescribers. However, there is still limited information about differences in provider responses to promotions for medications. In this study we investigated and evaluated strategies used by opioid manufacturers to encourage overprescribing, specifically focusing on oncology.

**Methods:**

We conducted a retrospective review of opioid industry documents released in litigation between 1999 and 2021. We began with a preliminary search for business plans in a subset of collections that identified key terms and phrases. These search terms were then used to narrow the investigation, which ultimately focused on Insys Therapeutics, and how they targeted oncology providers as well as patients with cancer pain.

**Results:**

We found that, overall, Insys sought to market to institutions with fewer resources, to less experienced and high-volume providers, and directly to cancer patients, with the goal of encouraging increased opioid prescribing and use.

**Conclusions:**

Our research revealed gaps in provider training that may make some providers more susceptible to pharmaceutical marketing. Developing and promoting continuing education courses for providers that are free from conflicts of interest, particularly at smaller institutions, may be one step towards reducing opioid overprescribing and its associated harms.

**Supplementary Information:**

The online version contains supplementary material available at 10.1186/s12889-024-19642-z.

## Introduction

Since 1999, the US has experienced at least three waves of opioid crisis, respectively involving prescription opioids, heroin, and synthetic opioids [1]. Although opioids have therapeutic value and are commonly used to treat moderate to severe cancer pain, [2, 3] more than 263,000 individuals died due to prescription opioid misuse between 1999 and 2020 [4, 5]. In 2013, prescription opioid use led to addiction in about two million people in the United States, resulting in an annual economic burden of $78.5 billion [6]. In 2021, the Congressional Research Service found that, despite regulatory efforts that reduced per capita US opioid consumption by 48% in 2009-19 [7], the US continued to have the highest rate of per capita opioid consumption in the world, primarily due to prescribing practices of US health care providers [8]. Providers in the US continue to prescribe opioids more often, prescribe at higher doses, prescribe higher-potency products, and prescribe them more commonly as a first-line treatment, than providers in other countries [8].

Since the 1990s, pharmaceutical companies have spent over $20,000 annually per physician to influence an increase in patented opioid prescriptions through detailing interactions with physicians [9]. Previous studies have found that the number of annual promotional visits by pharmaceutical sales representatives have been associated with a 13.3% increase in patented, or brand, opioid prescriptions and a 3.6% increase in generic opioid prescriptions [9]. This research also found an association between opioid marketing that directly targeted physicians and the level of opioid prescriptions. Between 2013 and 2015, pharmaceutical companies spent $39.7 million to encourage opioid prescribing by contacting over 67,000 physicians [10]. In 2014, among physicians who prescribed opioids under Medicare Part D, 7% received direct marketing payments from manufacturers that were associated with an increase in opioid prescriptions relative to those who did not receive any payments [11]. This marketing has been found to contribute to opioid overdose deaths [10].

There is known heterogeneity in prescribing in response to pharmaceutical industry promotions across provider specialties and geographical locations [9]. For example, family physicians and other high-contact providers such as internal medicine physicians have had a higher rate of prescribing in response to promotions than specialty providers such as surgeons [9]. However, there is still a gap in knowledge regarding the extent and source of the differences in provider responses to opioid marketing. Given the persistence of the opioid epidemic and disproportionately high opioid prescribing in the US, additional regulation of marketing strategies may be warranted, particularly in practice areas where opioid use is common such as oncology [11, 12]. Past regulatory proposals have suggested restricting direct-to-physician pharmaceutical marketing, limiting promotional payments to physicians, and requiring the inclusion of safety information and risks related to products in marketing materials [11, 13]. However, identifying appropriate proposed regulations has been limited by lack of information about the characteristics of marketing and the specific types of providers targeted by marketers.

Studying the potential effects of industry marketing on use of opioids has been challenging in part due to difficulties in identifying data that identifies how and why companies make marketing decisions [14, 15]. Research on other industries has addressed this limitation by reviewing internal documents released in litigation [15, 16] to identify industry marketing practices [15, 17–20]. These findings have been critical in generating changes in policy and practice that protect public health [15]. Recent lawsuits filed against the opioid industry have created extensive archives of internal industry documents that offer a unique perspective on the role of pharmaceutical companies in the on-going opioid crisis [16, 21]. In this study, we assessed activities of Insys Therapeutics, a specialty pharmaceutical company that marketed Subsys, a transmucosal immediate-release fentanyl (TIRF) spray targeted to patients experiencing breakthrough cancer pain who were already using opioid medications [22]. Subsys was the only opioid marketed by Insys, held a 16.1% market share of TIRFs in 2013, and generated a net revenue of $330 million in 2015 [22, 23]. In 2019, Insys executives were found guilty of racketeering conspiracy and the company settled criminal and civil charges made by the US Department of Justice by paying $225 million [24]. Although fentanyl, a synthetic opioid, is considered to have stronger effects than opiates such as heroin or morphine, in the US it is generally regulated comparably to other opioids [25]. The Insys case was considered a landmark in addressing the activities of pharmaceutical companies in “[marketing] opioids as an effective treatment for pain at all times” [26] and identifying how multiple pharmaceutical companies sought to increase sales “by any means necessary.” [26] We leveraged internal pharmaceutical industry documents released as part of the Insys litigation and settlement to evaluate the methods used to market opioids to providers seeking to treat cancer-related pain.

## Methods

We conducted a retrospective review of pharmaceutical industry documents released in litigation between 1999 and 2021. Research was conducted between January and May 2023. We retrieved documents contained in the Opioid Industry Document Archive (OIDA) at the University of California San Francisco (in collaboration with Johns Hopkins University) [27]. As of April 2023, OIDA held more than 3 million documents, containing more than 12.3 million pages and organized in 13 collections by source, that were made public during lawsuits against pharmaceutical companies, distributors, and pharmacies (link to the archive: https://www.industrydocuments.ucsf.edu/opioids [27]). These documents include emails, meeting and internal training presentations, spreadsheets on marketing budgets and sales performance, detailed business plans, public and government relations campaigns, trial transcripts, records of sales contacts, and details on the development and presentation of continuing education modules, among others. This research was conducted using an open access database available to the public; because it did not involve protected health information, it was excluded from institutional review board assessment.

## Search strategy

Given the size of the archive, we began searching for relevant documents with a preliminary search of three collections, containing 1,000 documents: Ohio Pharmacy Litigation Documents, Oklahoma Opioid Litigation Documents and Kentucky Opioid Litigation Documents. These collections were chosen specifically for their smaller collection sizes (228, 505, and 281 documents respectively) and yet allowed a reasonable capture of marketing themes across pharmaceutical companies in the initial review stage. Three authors with two years of doctoral pharmacy training (CL, AT, SX) each reviewed one of these collections, using common search terms across all three collections, tracking the terms used in a shared spreadsheet to identify common keywords. During this initial review, the search focused on business plans across pharmaceutical companies, as previous research using industry documents has found that this type of document often reveals marketing strategies, target audiences, and idiosyncratic terms used within companies to describe markets. The initial search terms included ‘business plan’, ‘plan’, ‘LTC [long-term care] AND “business plan”’, and ‘low back pain AND “business plan”’ (see Supplement). Initial search terms such as ‘LTC’ were drawn from previous research using opioids documents, which identified older adults as a target market for opioid promotions [28].

This initial search generated common themes across business plans that drove further exploration. Terms such as low back pain, neuropathy pain, cancer pain, and breakthrough cancer pain (BTCP) were repeatedly identified in opioid marketing. Prescribers’ specialties mentioned repeatedly in these initial searches included oncology, primary care, geriatrics, and anesthesiology. Settings identified include skilled-nursing facilities (SNFs) and hospice or elderly care. Although our initial searches were built using terms related to older adults given prior research identifying industry interest in this group, [28] oncology-related terms appeared the most frequently, and as a result, further searches concentrated in this area.

The key search terms identified in the review of the initial three collections were then used to search the Insys Litigation Documents Collection of more than 1.5 million documents; we chose this collection because it contained the most documents related to oncology marketing. Terms used for searching the collection included ‘oncology’, ‘strategy’, ‘sales’, ‘cancer’, and ‘region’ (see Supplement). Documents such as trial transcripts were excluded in the search as they were found to be duplicative of original documents produced by companies. Each of the three researchers involved in searching (CL, AT, SX) focused on a subset of these key terms and documented the results in a shared spreadsheet.

## Analytical approach

Documents were listed with summary information and discussed by all four authors in weekly meetings. During these meetings, documents identified as potentially relevant by one researcher were reviewed by a second person for validation. Potentially relevant documents included communication records that did not explicitly state their marketing tactics, but rather hinted at them, while documents that were considered relevant were either those that authors believed certain implications could be made, or those that marketing tactics were explicitly stated. Documents confirmed as relevant were then discussed by the group and categorized into key themes that were consistent across documents, companies, and collections. Themes were identified inductively from review of the materials and based on the study objective of identifying marketing strategies specific to different types of providers. Documents that reported the use of similar marketing strategies were categorized together, while new categories were created for documents presented with new ideas and themes. When there was uncertainty regarding how a document should be categorized, it was marked for review and discussed by all authors, including one with over 20 years of industry documents research experience and over 5 years of experience studying pharmaceutical industry marketing (DA), until a consensus was reached regarding its relevance and appropriate categorization. Documents were ultimately categorized based on the following themes: institution type, individual providers, prescribers, patients, and other. Examples of documents in the “other” category included geographical analysis of sales territories and choices of media advertisements. There were too few of these relevant documents to justify the creation of additional categories, although they nonetheless had implications with respect to strategies used to encourage opioid overprescribing.

### Patient and public involvement

Patients or the public were not involved in the design, or conduct, or reporting, or dissemination plans of our research.

## Results

Our final analysis relied on 21 key documents that detailed the primary strategies used by Insys Therapeutics to target oncology providers and increase opioid sales. Based on the email communication within the sales teams, performance reports, presentation materials, and business plans, we identified three common areas where the company sought to incentivize increased opioid prescribing: (1) institutions with fewer resources and that were densely co-located, (2) less experienced and high-volume providers, and (3) cancer patients and related advocacy groups.

### Institutions with fewer resources and that were densely co-located

**Fig. 1:**
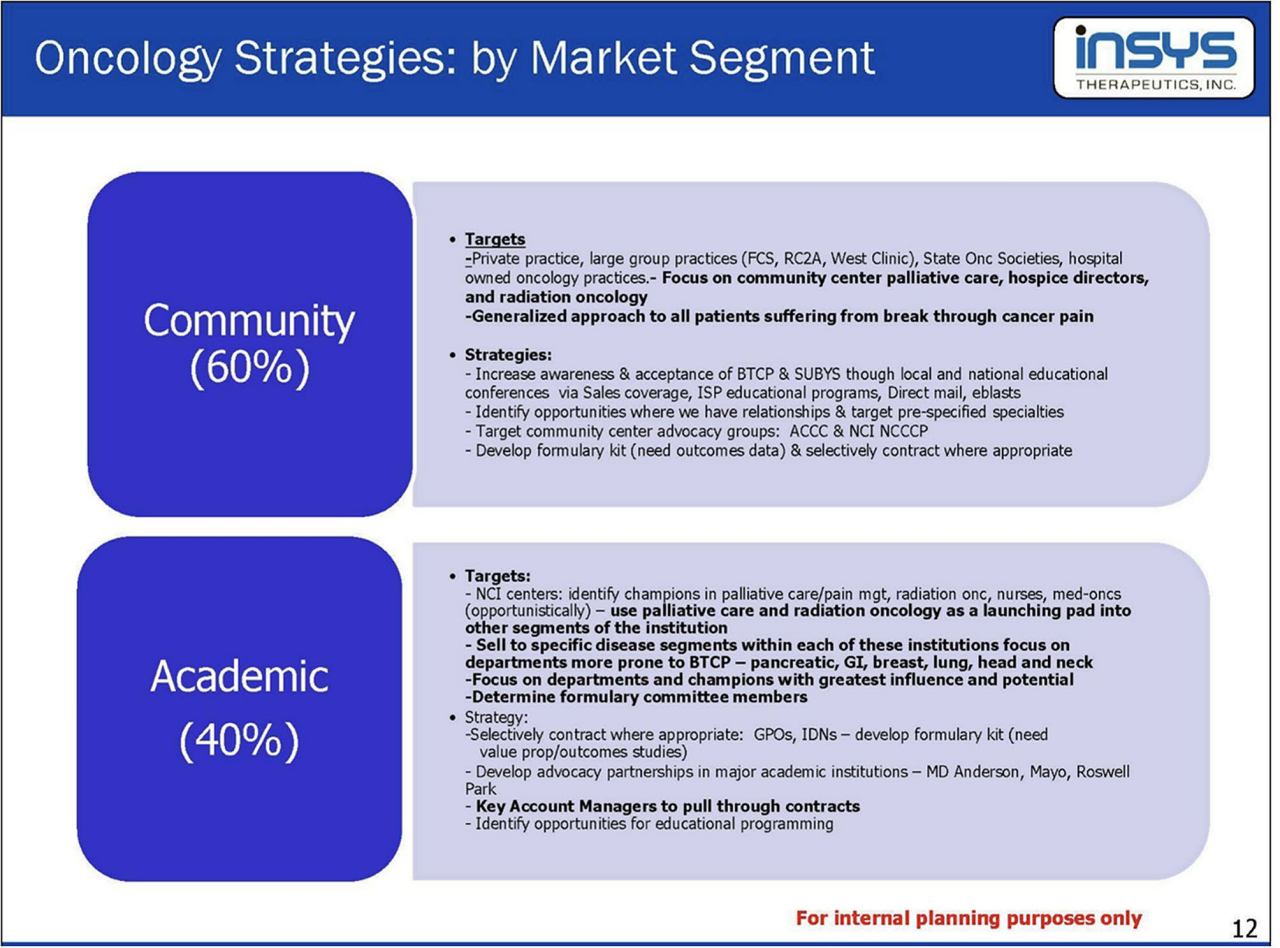
Excerpt from August 2014 “Oncology Strategy Meeting”. Presentation was from an internal planning presentation by Insys Therapeutics to demonstrate how representatives can differ their strategies based on the type of institution: -- community and academic. Text: “Community (60%) Targets: Private practice, large group practices (FCS, RC2A, West Clinic), State Onc Societies, hospital owned oncology practices – Focus on community center palliative care, hospice directors, and radiation oncology. Generalized approach to all patients suffering from break through cancer pain. Strategies: Increase awareness & acceptance of BTCP & SUBSYS through local and national educational conferences via Sales coverage, ISP educational programs, Direct mail, eblasts. Identify opportunities where we have relationships & target pre-specified specialties. Target community center advocacy groups: ACCC & NCI NCCCP. Develop formulary kit (need outcomes data) & selectively contract where appropriate. Academic (40%) Targets: NCI centers: identify champions in palliative care/pain management, radiation onc, nurses, med-oncs (opportunistically) - use palliative care and radiation oncology as launching pad into other segments of the institution. Sell to specific disease segments within each of these institutions focus on departments more prone to BTCP – pancreatic, GI, breast, lung, head and neck. Focus on departments and champions with greatest influence and potential. Determine formulary committee members. Strategy: Selectively contract where appropriate: GPOs, IDNs – develop formulary kit (need value prop/outcomes studies). Develop advocacy partnerships in major academic institutions – MD Anderson, Mayo, Roswell Park. Key Account Managers to pull through contracts. Identify opportunities for educational programming.” Source: https://industrydocuments.ucsf.edu/opioids/docs/#id=tzhm0278

In 2012, Insys Therapeutics launched Subsys, a fentanyl sublingual spray, for breakthrough pain in patients with cancer who are opioid tolerant [24]. Documents within the company detailed that they specifically sought to market to healthcare providers who specialized in oncology, encouraging them to treat breakthrough cancer pain. In 2014, Insys hosted an Oncology Market Segments training for internal planning, in which they proposed marketing strategies for both academic and community settings (Fig. 1) [29]. Within the academic setting, they focused on specific cancers identified as being more prone to breakthrough cancer pain, while also targeting influential champions and members of formulary committees [29]. Champions were defined as individuals working in healthcare who engaged with new initiatives during product development [30]. Within the community setting, Insys pursued a primary strategy of discussing generalized breakthrough cancer pain with community palliative care centers, hospice directors, and radiation oncologists [29]. Insys targeted different individuals to help establish relationships within practices depending on setting. Within institutions, the company targeted prescribers working in palliative care [31]. In community settings, Insys primarily targeted nurses and “mid-levels”, [31] defined as healthcare providers who could practice and provide high-quality care independently, but were considered to have less training than physicians, such as nurse practitioners and physician assistants [32].

**Fig. 2:**
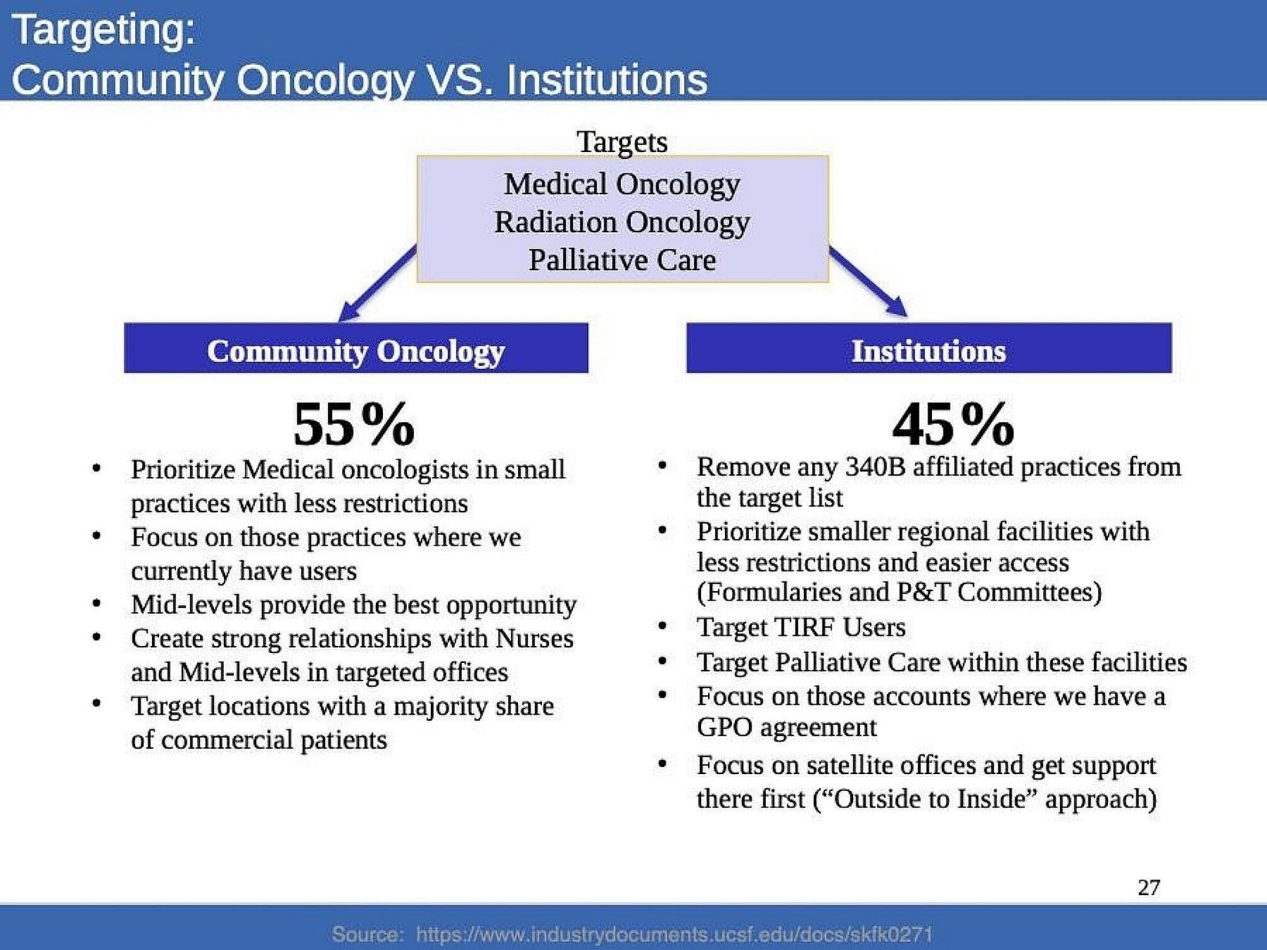
Excerpt from the February 2012 “Radiation Oncology Strategy Discussion” by Insys Therapeutics. Example of how Insys tailored their strategies depending on if the healthcare provider practiced in a community oncology setting or an institution. Text: “Targets: Medical Oncology, Radiation Oncology, Palliative Care. Community Oncology 55%: Priorite Medical oncologists in small practices with less restrictions. Focus on those practices where we currently have users. Mid-levels provide the best opportunity. Create strong relationships with Nurses and Mid-levels in targeted offices. Target locations with a majority share of commercial patients. Institutions 45%: Remove any 340B affiliated practices from the target list. Prioritize smaller regional facilities with less restrictions and easier access (Formularies and P&T Committees). Target TIRF Users. Target Palliative Care within these facilities. Focus on those accounts where we have a GPO agreement. Focus on satellite offices and get support there first (“Outside to Inside” approach).” Source: https://industrydocuments.ucsf.edu/opioids/docs/#id=tzhm0278

In 2014, Insys Therapeutics also hosted an internal planning session titled “Advocating for People with Breakthrough Cancer Pain.” [33] The objective of this meeting was to establish Subsys as the leader for symptom management in breakthrough cancer pain [33]. The marketing plan primarily focused on oncologists practicing in the community setting, as the company hypothesized that they would be less likely to have access to additional supportive care specialists, such as oncology nurses, palliative care experts, and pain specialists [33]. As a result, the company anticipated that these providers would be more receptive to prescribing Subsys as a primary means of pain management [33]. In 2015, Subsys also hosted a meeting called, “Radiation Oncology Strategy Discussion,” where they described the radiation oncology profession and potential strategies to target these specific healthcare providers [31]. They emphasized that community centers were likely to have fewer restrictions on prescribing that might limit use of Subsys [31]. For academic institutions, the company encouraged sales representatives to target smaller organizations that would have fewer prescribing restrictions in place [31]. A slide from this presentation is included in Fig. 2.

**Fig. 3:**
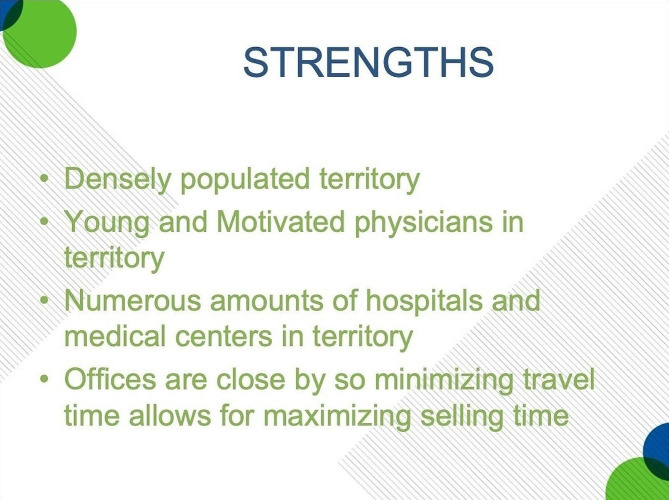
Excerpt from January 2014 “Built to Last” Meeting. Example of geographical considerations while identifying areas to target. Text: “Strengths: Densely populated territory. Young and Motivated physicians in territory. Numerous amounts of hospitals and medical centers in territory. Offices are close by so minimizing travel time allows for maximizing selling time.” Source: https://industrydocuments.ucsf.edu/opioids/docs/#id=nxph0276

In addition to the type of institution, Insys considered geographical factors. In a 2014 “Built to Last” meeting, Insys discussed areas, as well as customers, their company had targeted thus far. Desirable factors considered included population density, the type of providers who practiced in the area, the number of healthcare institutions within the vicinity, as well as the distance between institutions that would maximize sales efficiency [34]. A slide from this meeting is included in Fig. 3.

### Less experienced and high-volume prescribers

Throughout the archive, different opioid manufacturers targeted providers who they believed were less experienced and well-educated regarding pain management with opioids [35]. Providers identified in this category included primary care physicians, because they have a wide range of responsibilities, and Insys anticipated there was less likelihood that they emphasized pain care in their practices [35]. The United States General Accounting Office conducted a study in 2003 to review the rapid sales growth of OxyContin in early 2000s, which noted that by 2003, half of all OxyContin prescribers were primary care physicians [35]. In the same year, the US Drug Enforcement Administration (DEA) specifically noted concern about aggressive marketing to physicians who might not be adequately trained in pain management [35].

**Fig. 4:**
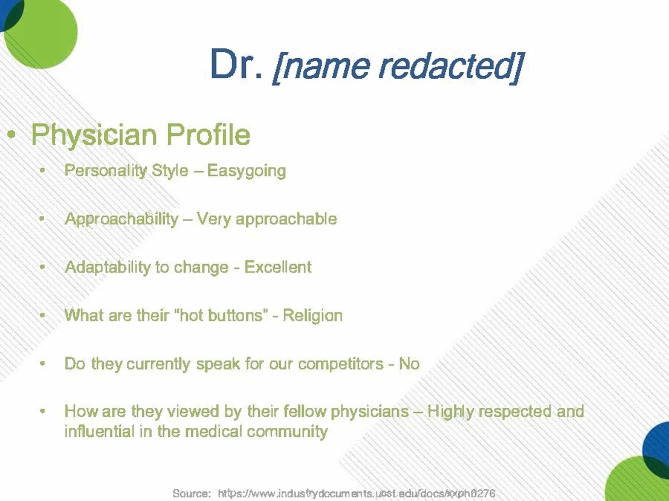
Excerpt from January 2014 “Built to Last” Meeting. Presentation shows how Insys Therapeutics would profile potential physicians to identify individuals to continue targeting or those to recruit as potential speakers for future bureaus and conferences. Text: “Dr. [name redacted] Physician Profile: Personality Style – Easygoing. Approachability – Very approachable. Adaptability to change – Excellent. What are their ‘hot buttons’ - Religion Do they currently speak for our competitors – No. How are they viewed by their fellow physicians – Highly respected and influential in the medical community.” Source: https://industrydocuments.ucsf.edu/opioids/docs/#id=xxph0276

When Insys Therapeutics launched Subsys in 2012, sales representatives within the company identified lists of providers that they believed would be most likely to prescribe it [36]. Sales representatives then provided updates on specific individuals including their interests, concerns, potential barriers to their increased prescribing, and their plans to increased prescribing (see Fig. 4) [36]. A common strategy for prescriber outreach was to host lunch or dinner programs, partly because some prescribers would only consider meeting sales representatives over meals [36, 37]. In order for sales representatives to build relationships with new prescribers and office support staff, Insys budgeted for sales representatives to provide free lunches to providers averaging $20–25 per person, from at least 2012 to 2015 [38]. To facilitate the prescribing process, representatives also worked with these physicians’ preferred pharmacies to enroll them into the TIRF REMS programs and to stock Subsys [36].

In addition to free meals, Insys developed speaker programs intended to attract more healthcare providers to become new prescribers. In the 2013 Insys Therapeutics “Proposed Marketing Budget” for Subsys, $3.3 million was allocated to Medical Communication, representing 71% of the total budget [39]. Of that, $2.3 million was designated to the ‘Insys Speaker Program’ [39]. The company used speaker programs to generate peer-to-peer dialogues and gauge interest from healthcare providers who would potentially prescribe Subsys [38]. Speakers had clinical experience with Subsys and were trained by the company to promote it [40]. Their first-hand prescribing experience was expected to make Subsys use more compelling to prescribers who had not prescribed it in the past and were hesitant about starting [40].

Sales representatives from pharmaceutical companies also joined national, regional, and local conferences to connect with groups of providers in order and recruit them to endorse medications [41]. In 2014, sales representatives hosted exhibits and activities at the Oncology Nursing Society (ONS) Congress, the largest oncology nursing conference in the US, attended by thousands of nurse professionals [42]. Attendees’ contact details were collected and added to the companies’ customer database, allowing sales teams to identify prescribers with “high potential” and follow up immediately [43].

In addition to new prescribers, Insys encouraged increased prescribing of opioids by providers who were already high-volume prescribers. After identifying these high-volume prescribers, Insys invited them to present at speaker programs [44, 45]. The company maintained a “Speaker Bureau” for which they created an “invitation list” that included providers in highest deciles of prescribing [46]. In a 2016 internal analysis, Insys indicated that it had spent $40,000 to $360,000 on speaker programs each month, with 70% of total spending on honoraria, largely directed to high-volume prescribers [45]. Amounts paid varied by specialty and the speakers’ perceived status in the community, with nationally recognized providers receiving higher payments than those with only regional recognition [47]. Insys also preferred speakers with an interest in symptom management (such as those with a membership in pain care forums) [33]. Honoraria were also based on the number of programs providers attended [45]. Oncologists typically received the most generous honoraria [47].

The company also advertised in professional healthcare journals in an effort to reach new providers [48, 49]. During one of the Subsys Board of Directors Meeting in July 2014, Insys noted that it was using journal advertisements to target oncologists and nurses [49]. The company’s Oncology Steering Committee meeting in August 2014 indicated that advertising in professional journals would increase name recognition [48]. Targeted journal types included oncology, hospice, and palliative care, [50] including the American Society of Clinical Oncology Post (ASCO Post), Journal of Clinical Oncology (JCO), and Journal of Clinical and Supportive Oncology [51].

### Cancer patients and related advocacy groups

**Fig. 5:**
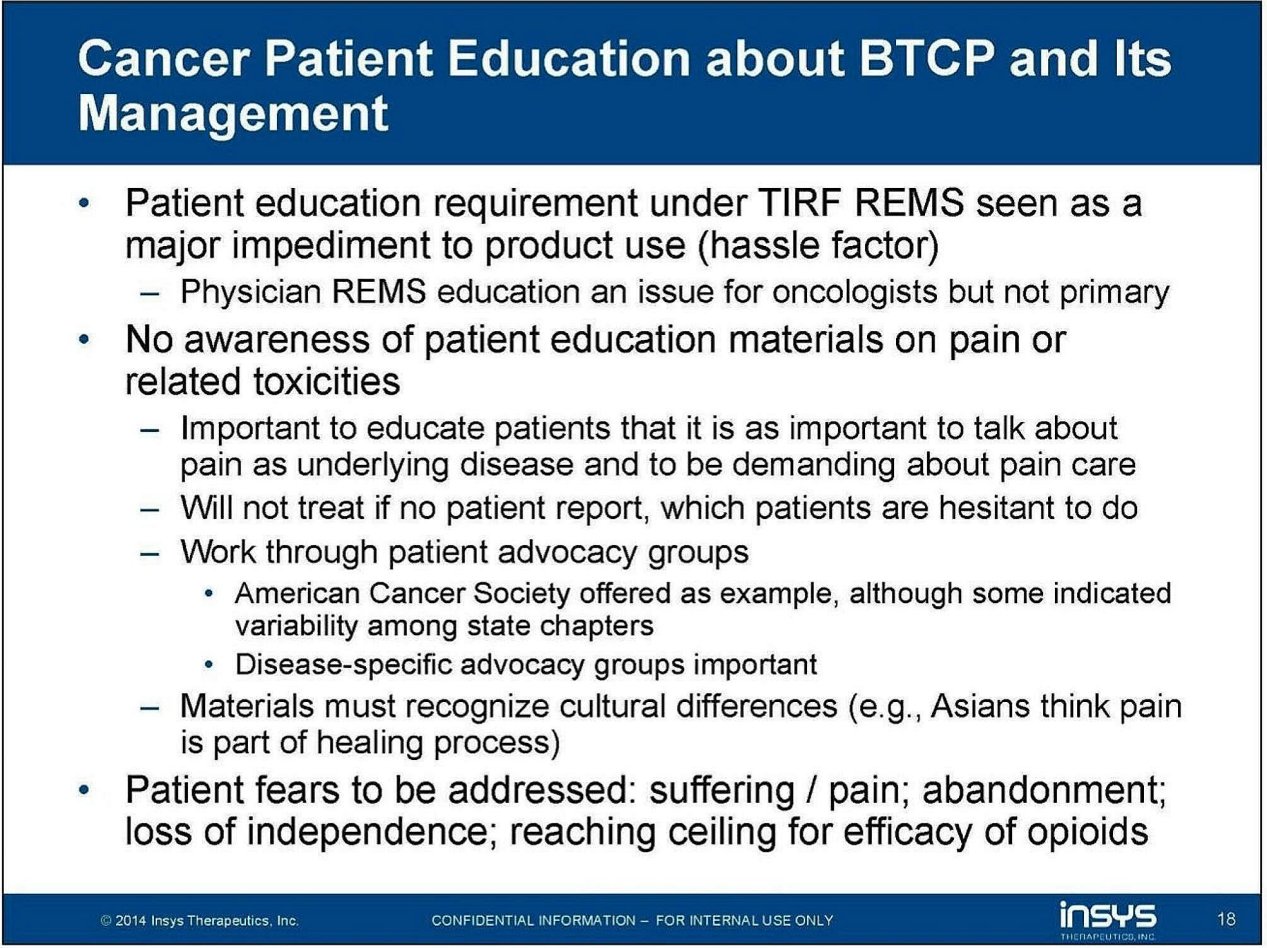
Excerpt from the 2014 Insys Therapeutics “Oncology Steering Committee”. Examples of strategies employed to directly outreach to patients for BTCP management education. Text: “Cancer Patient Education about BTCP and Its Management. Patient education requirement under TIRF REMS seen as a major impediment to product use (hassle factor). Physician REMS education an issue for oncologists but not primary. No awareness of patient education materials on pain or related toxicities. Important to educate patients that it is as important to talk about pain as underlying disease and to be demanding about pain care. Will not treat if no patient record, which patients are hesitant to do. Work through patient advocacy groups. American Cancer Society offered as example, although some indicated variability among state chapters. Disease-specific advocacy groups important. Materials must recognize cultural differences (e.g., Asians think pain is part of healing process). Patients feared to be addressed: suffering / pain; abandonment; loss of independence; reaching ceiling of efficacy of opioids.” Source: https://industrydocuments.ucsf.edu/opioids/docs/#id=nmgm0278

Insys also made attempts to directly communicate with patients. In 2014, the company hosted an Oncology Steering Committee meeting, where they developed patient educational materials [48]. One main emphasis was encouragement for patients to, “be demanding about pain care.” [48] The company developed additional materials that addressed what it claimed were cultural differences, for example, “Asians think pain is part of healing process.” (Fig. 5) [48] Insys also sought to collaborate with patient advocacy groups [33, 48]. When considering which support groups to target, the company considered previous demonstrations of interest in symptom management (in the form of memberships in pain care forums or web-based information); whether the patients represented by an organization were diagnosed with common tumor types, and the type of public recognition groups had established [33].

## Discussion

Our results suggest that Insys focused its opioid marketing on institutions perceived to have fewer resources and restrictions on prescribing, less-experienced providers, and high-volume prescribers, as well as encouraging cancer patients and advocacy groups to demand opioids for pain management. It targeted institutions with fewer resources anticipating that they had limited existing knowledge or experience in breakthrough cancer pain management, and making them more receptive to resorting to opioids [33]. This marketing strategy explicitly sought to target community centers where institutional knowledge was less extensive, and where there were fewer procedures in place to assess whether opioid prescriptions were appropriate, in order to increase opioid prescribing [33]. The FDA’s Adverse Event Reporting System (FAERS) reported over 750 adverse events related to Subsys, with three-quarters of those involving deaths [52]. The US Department of Justice identified Insys marketing as responsible for opioid overprescribing in vulnerable patient populations, which likely contributed to health harms associated with the opioid epidemic [52].

Insys also targeted prescribers with less experience, in part by paying providers open to high-volume prescribing to advocate for use of Subsys [40]. This peer-to-peer approach was intended to establish trust in the product by linking the medication to colleagues that were perceived to be reputable. This strategy was expected to be particularly effective with providers who had less experience; given that these prescribers included nurse practitioners, which did not have to disclose promotional payments from pharmaceutical companies until 2021, [53, 54] this group may also have been targeted as a strategy to limit public awareness of marketing activities. In addition to recruiting speakers, Insys developed continuing education programs and paid for meals to advertise Subsys to providers. This included providing honoraria to high-volume prescribers to continue encouraging opioid prescriptions. It is possible that prescribers recruited as speakers felt pressure to maintain a high level of opioid prescribing to demonstrate clinical experience. This could potentially lead to inappropriate prescribing for patients that did not need such opioids and increase their risk for opioid dependency and abuse. Finally, Insys encouraged cancer patients to seek opioids as treatment, especially for breakthrough cancer pain. Historically, pharmaceutical companies have targeted patient advocacy groups to both promote their products and garner legal support through donations. As a result, when the Center of Disease Control and Prevention (CDC) drafted guidelines to further restrict opioid prescribing in 2016, organizations with relationships with opioid manufacturers lobbied against the policies and created their own guidelines to give little weight to opioid addiction risks [55].

Our study has limitations. As an observational study, it cannot establish causality or account for missing factors. Although OIDA contained over 3 million documents as of April 2023, the archive does not contain all documents produced by pharmaceutical companies that manufactured, sold, or dispensed opioids, meaning that the findings represent an incomplete record of opioid marketing strategies. In addition, for some documents, companies redacted information that they claimed revealed trade secrets. Many of the documents included in the archive were strategic plans or budgets; it is possible that not all corporate plans were executed. Although we documented our searches and analytical strategy, the categorization and analysis were based on the interpretations of the authors. Our research considered documents specific to Insys Therapeutics, which may not necessarily be generalizable to other companies given the focus on a single company. Future research could investigate whether the marketing efforts we identified, such as attempting to influence less experienced providers, are consistent for other companies and other types of medications, and whether these efforts have continued. In addition, our focus on promotion of Subsys to treat cancer pain did not assess off-label promotion and uses of this medication; future research could consider the extent to which this is described in the Insys documents.

Despite these limitations, internal industry documents provide a unique and contemporaneous perspective on the marketing strategies used by opioid manufacturers that would be difficult or impossible to identify using other sources and identified patterns that may inform interventions to reduce excessive opioid prescribing and improve public health.

### Conclusions and implications

Much of the previous research based on opioid industry documents has examined how marketing strategies of pharmaceutical companies affected policymakers and consumers, as well as suggesting that providers were targeted [28, 56]. This research builds on these findings by identifying key characteristics of providers and institutions that were targeted to increase opioid prescribing: specifically, Insys marketed Subsys by marketing to organizations with fewer resources and institutional guidelines that would have prevented inappropriate opioid prescribing, as well as to providers and patients that lacked experience to assess the accuracy of corporate marketing claims. They may continue to be vulnerable to these opioid marketing strategies, and companies seeking to market other addictive medications may pursue similar strategies. In addition, Insys provided direct financial support to high-volume prescribers to encourage them to continue suggesting opioid use. Taken as a whole, these findings reveal gaps in provider training and in regulation of opioid industry activities that could potentially be addressed by providing shared resources and common prescribing guidelines for smaller healthcare institutions and requiring some or all continuing education courses to be provided by unconflicted presenters.

### Electronic supplementary material

Below is the link to the electronic supplementary material.

Supplementary Material 1

## Data Availability

The data supporting the conclusions of this article are publicly available at the University of California Industry Documents Library. https://www.industrydocuments.ucsf.edu/.
