# Supplementary material for: Evaluation of the strategies opioid manufacturers used to recruit health professionals and encourage overprescribing: an analysis of industry documents
Source: BMC Public Health. 2024 Aug 8;24:2153. doi: 10.1186/s12889-024-19642-z (PMC11308442; doi:10.1186/s12889-024-19642-z)
Supplement: Supplementary file 1 — Supplementary Material 1 [file 12889_2024_19642_MOESM1_ESM.docx]

# Evaluation of the strategies opioid manufacturers used to recruit health professionals and encourage overprescribing: an analysis of industry documents

## Supplement

## Table S1: Search terms; searches were conducted between April 6^th^, 2023 – May 7^th^ 2023.

Source: Data collected by the authors

| **Search Term** | **Filters Applied** | **# of Results** |
| --- | --- | --- |
| LTC AND “business plan" | [refer to Sheet 2 for detailed search results] | 122 [note: only the first 9 results are the actual business plans] |
| plan | Oklahoma collection | 248 |
| plan | Oklahoma collection, type: presentation | 13 |
| oncology AND strateg*  -> Teva payments to Advocacy Groups  -> More like this: Teva Advocacy Mapping: Identifying Advocacy Partners to Enhance Patient Care | Oklahoma collection | 62 |
| oncolog* and "strateg*" | No active filters | 117 |
| oncology AND strateg* | Oklahoma collection | 62 |
| oncology AND strateg*  -> Teva payments to Advocacy Groups  -> More like this: Opioid Manufacturer Payments to Advocacy Organizations Compared to Teva (2012-2017) | Oklahoma collection | 62 |
| oncology | Kentucky collection | 4 |
| oncology | Kentucky collection | 4 |
| oncology AND region AND sales => oncology programs/lunches => more like this: RE: AAMC ONCOLOGY AND HEMATOLOGY - 2016 LUNCHES | No active filters | 4,535 |
| oncolog* AND plan | No active filters | 23,042 |
| oncology AND region AND sales | Spreadsheet | 171 |
| primary care AND strateg* | Kentucky collection | 18 |
| cancer AND strateg* | Kentucky collection | 15 |
| (steven chun)  Note: identified the name from Insys' Top HCP Spreadsheet (refer to Sheet 2) | No active filters | 23 |
| (steven chun) | No active filters | 23 |
| oncolog* | Type: Presentation | 2,658 |
| oncolog* | Type: Presentation | 2,658 |
| oncolog* | Type: Presentation | 2,658 |
| oncolog* AND territor* | Type: Presentation | 444 |
| honorari* AND oncolog* | None | 2,311 |
| honorari* AND oncolog* | None | 2,311 |
| honorari* AND oncolog* | None | 2,311 |
| (Daniel Tondre)  Note: identified the name as Steven Chun's SSP, also one of the top SSPs | None | 12,230 |
| (Daniel Tondre) AND (Steven Chun) | Email | 292 |
| (Daniel Tondre) AND (Steven Chun) | Email | 292 |
| "Steven Chun" | Email | 2,694 |
| "speaker bureau" | Insys collection | 6,338 |
| "Oncology Nursing Society" | Email | 325 |
| "BTCP" | Presentations | 2,467 |
| "BTCP" | Presentations | 2,467 |
| "BTCP" | Presentations | 2,467 |
| "BTCP" | Presentations | 2,467 |
| "BTCP" | Presentations | 2,467 |
| Amer K. Syed | No active filters | 108 |
| Amer K. Syed | No active filters | 108 |
| anesthesiolog* AND prescriber* | Email | 1,011 |
| oncolog* AND lunch AND "new prescriber" | Email | 28 |
| "oncology nursing society" AND "ancillary" | No active filters | 116 |
| "oncology nursing society", found from more like this - RE: HOT REQUEST: ONS Ancillary Event (prym0268) | No active filters | N/A |
| journal ads | Insys, email | 698 |
| journal ads | Insys, email | 698 |
| journal advertisements | Insys | 150 |
